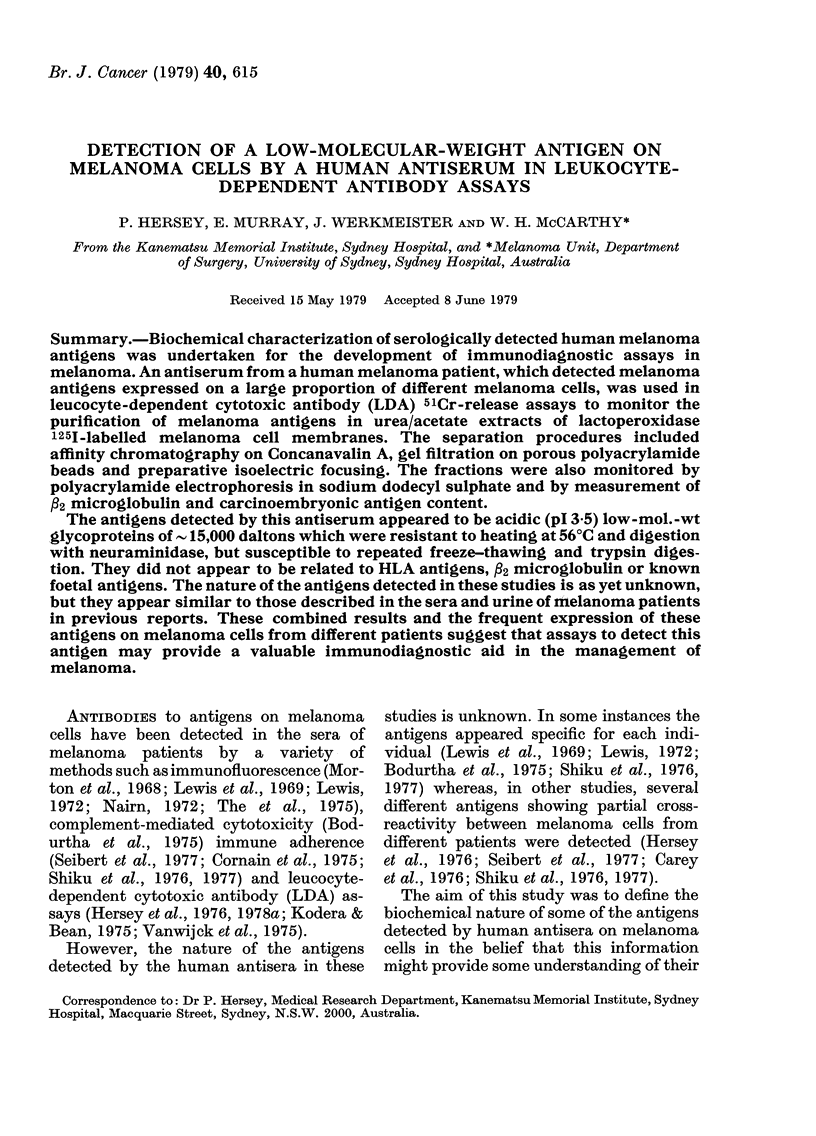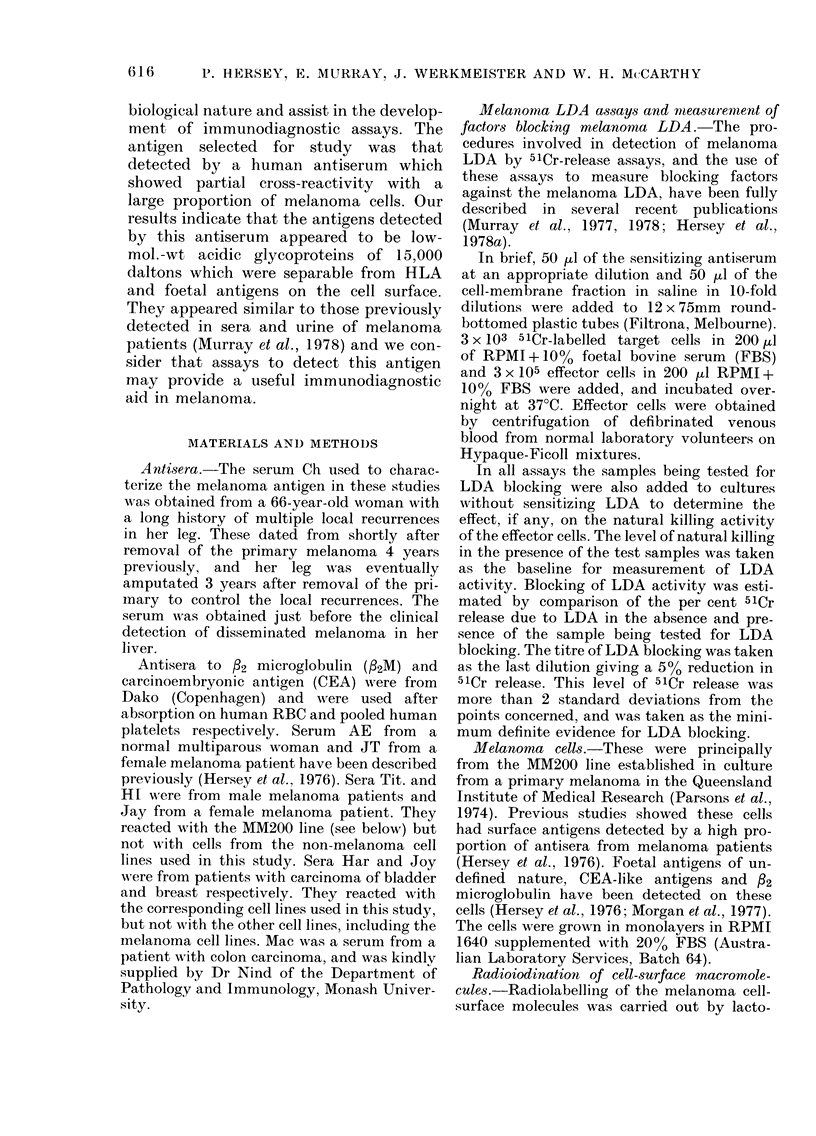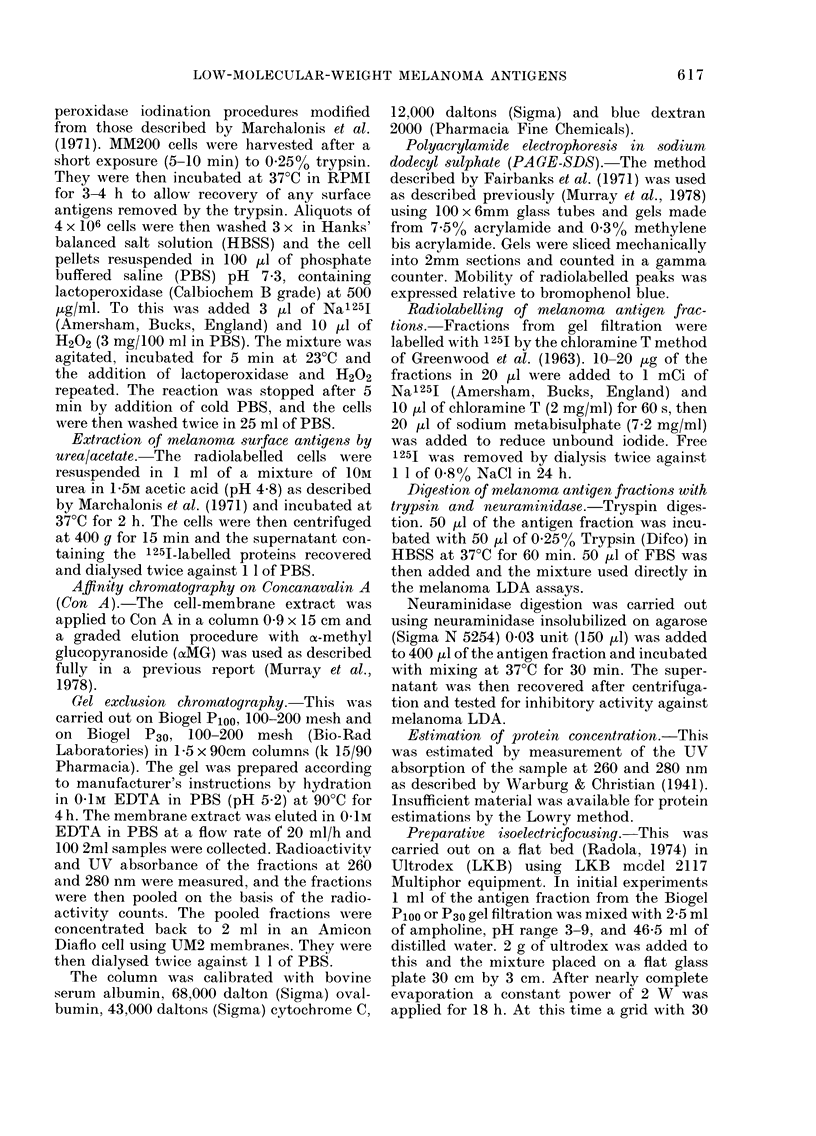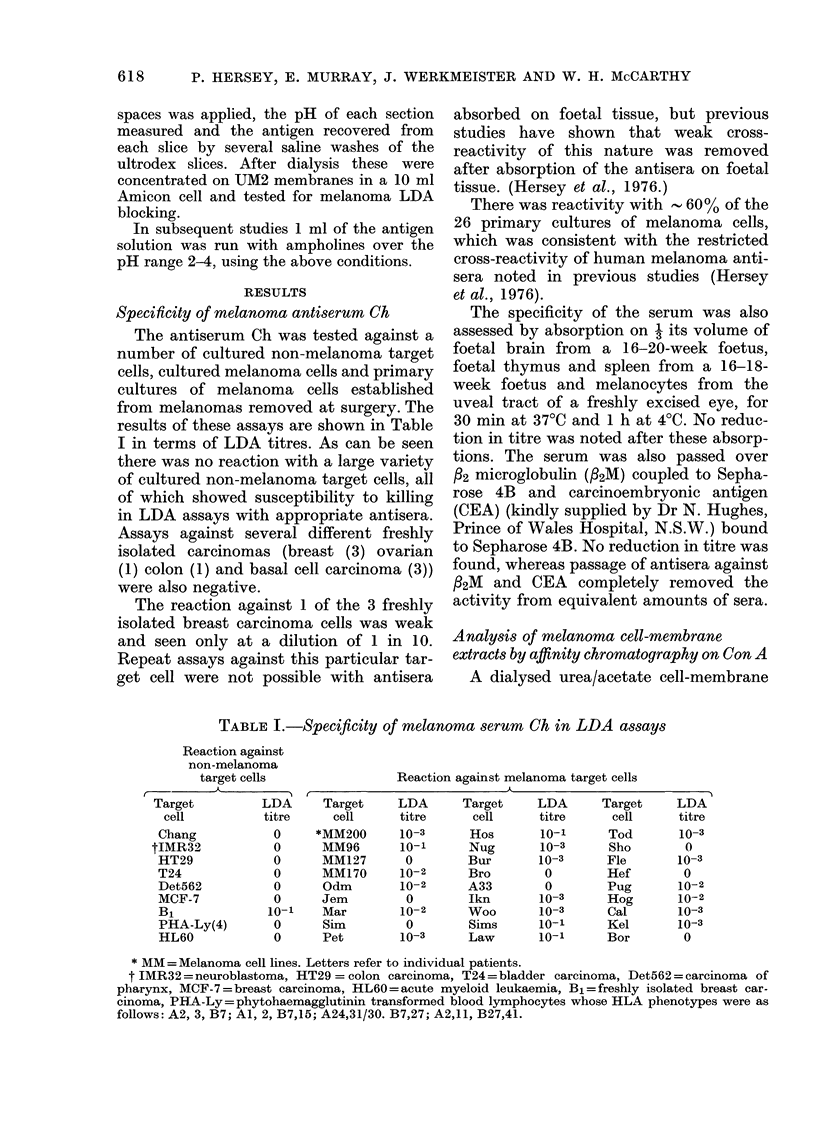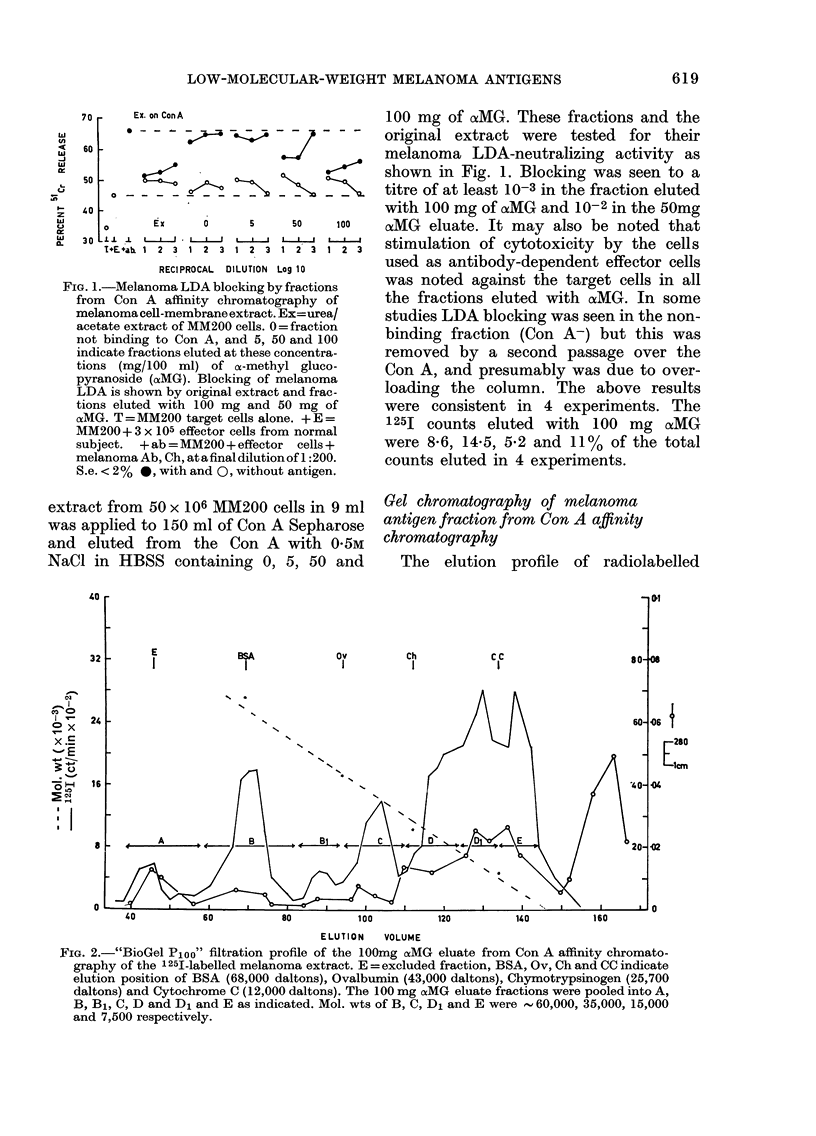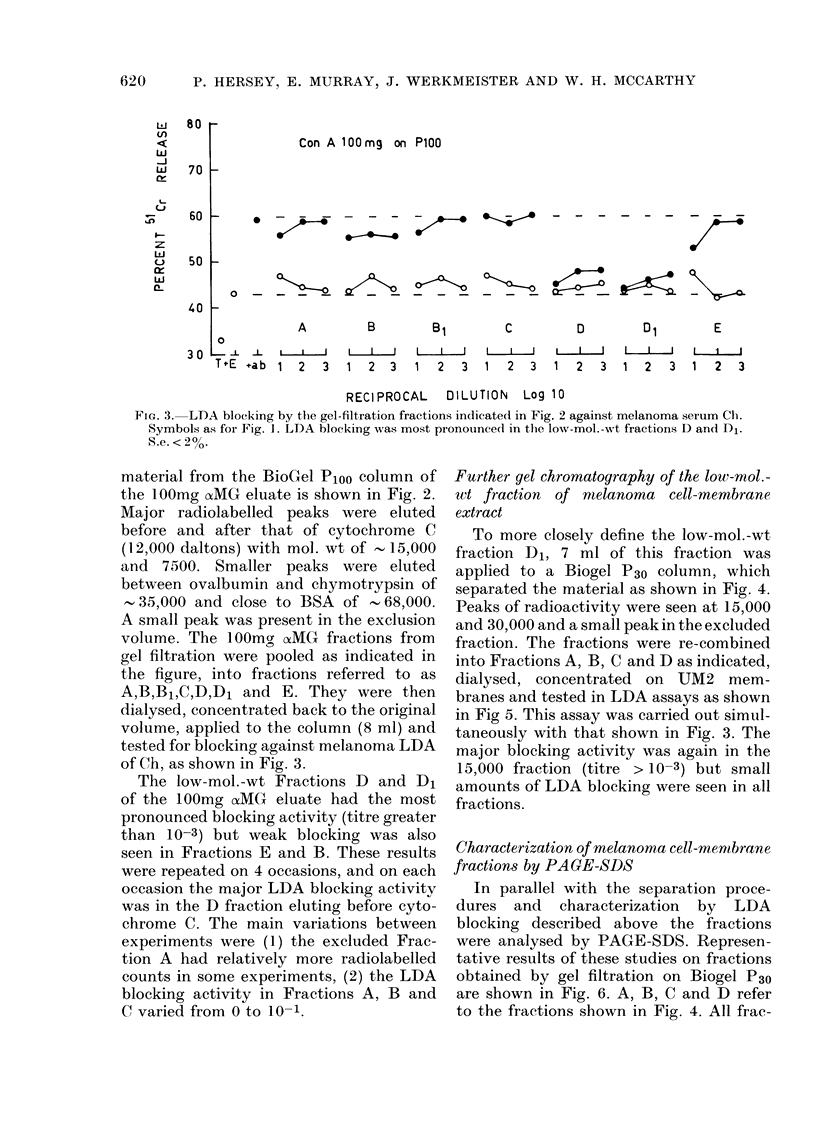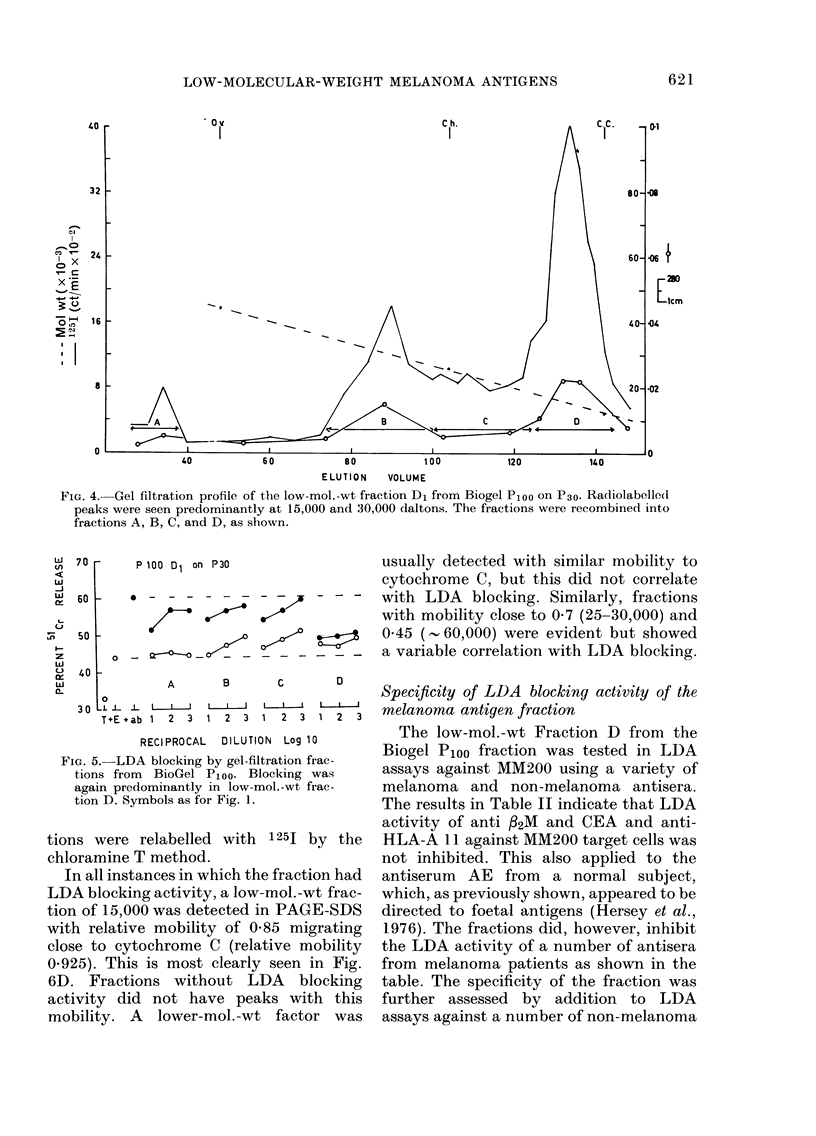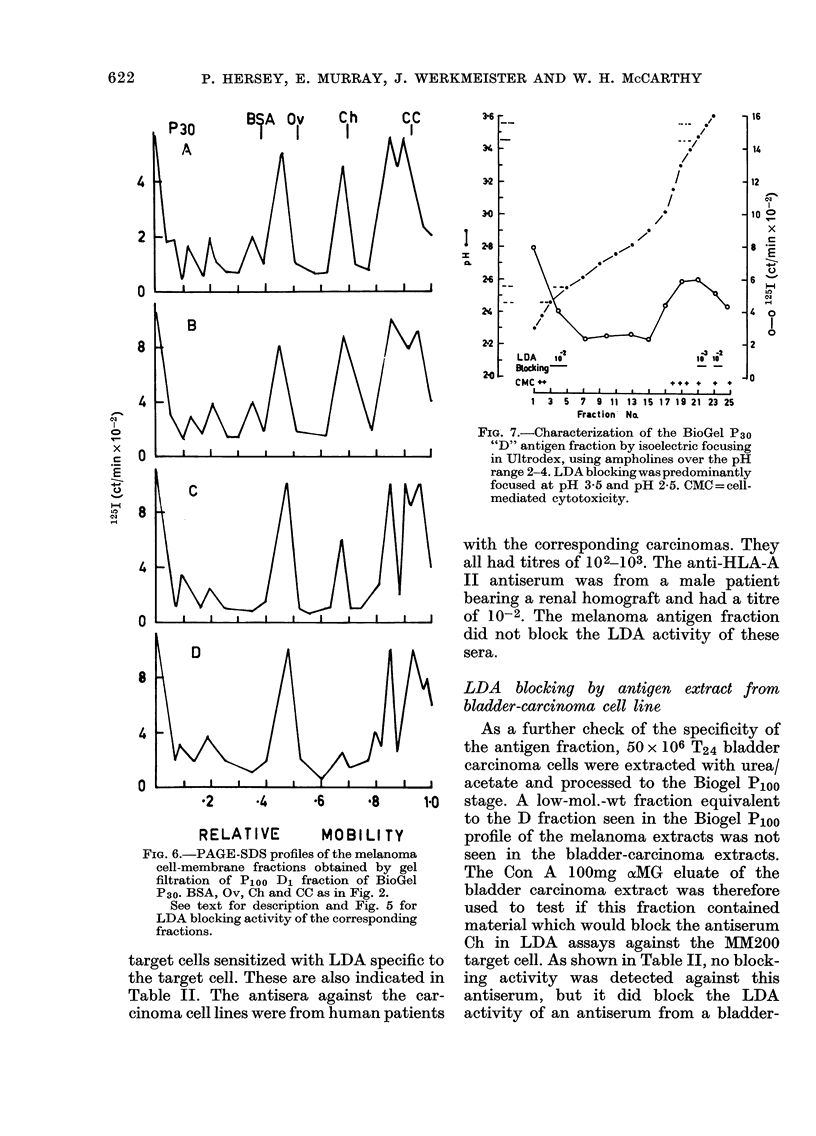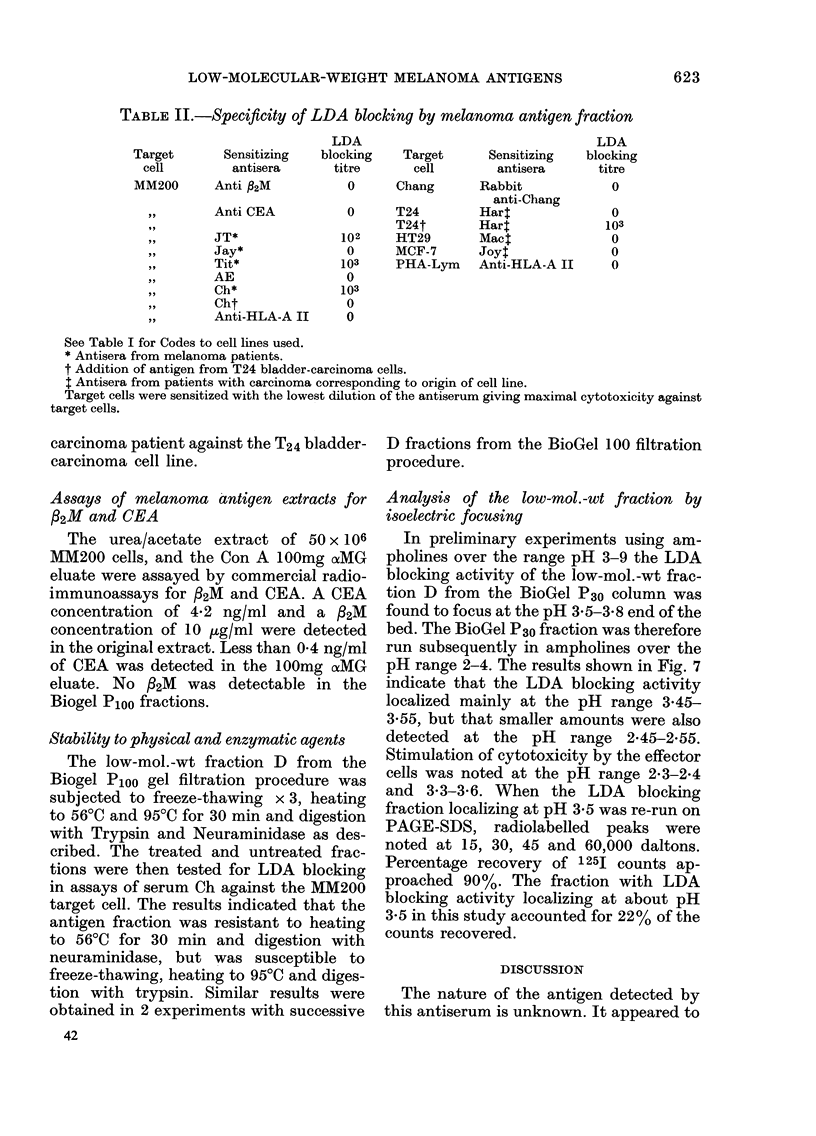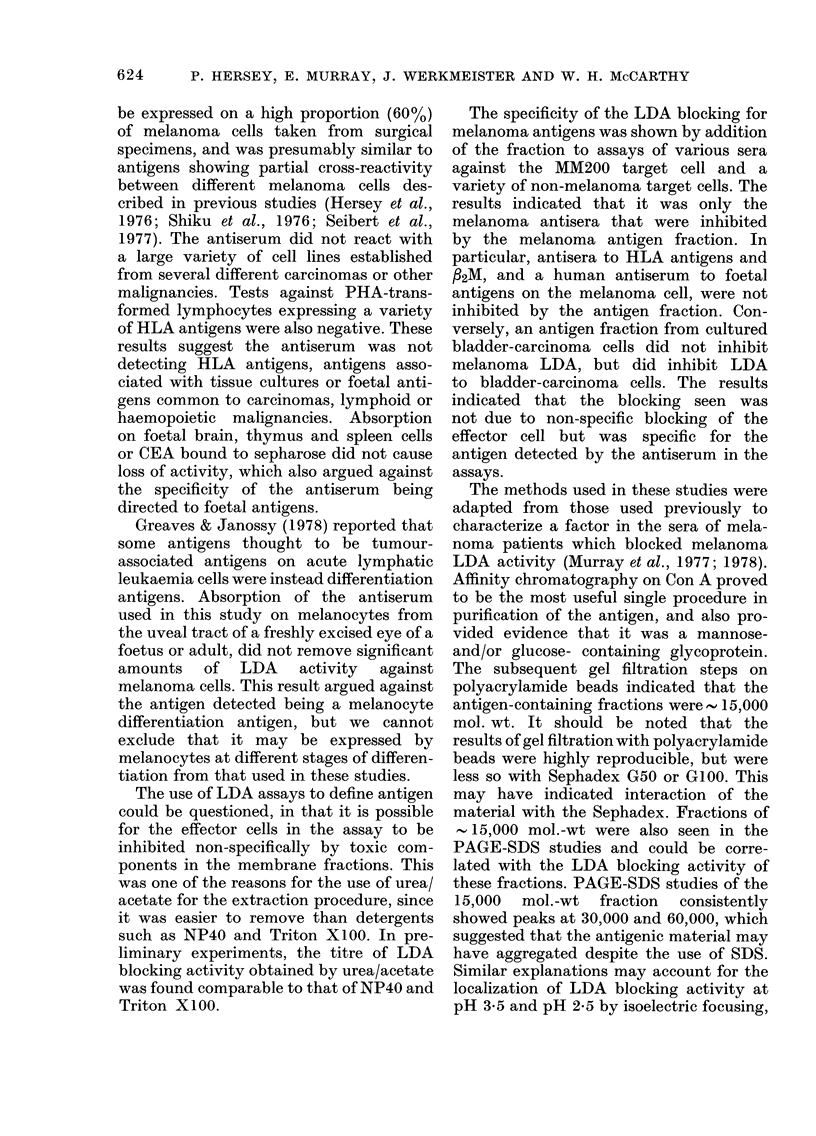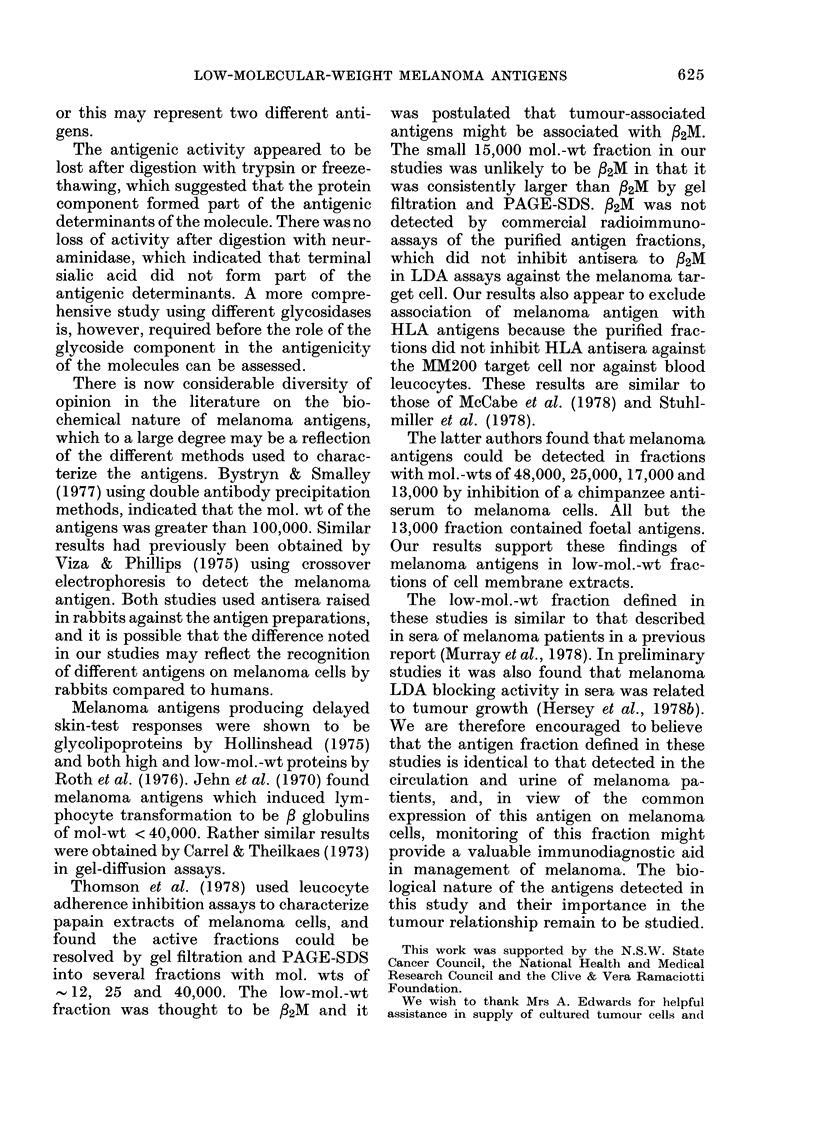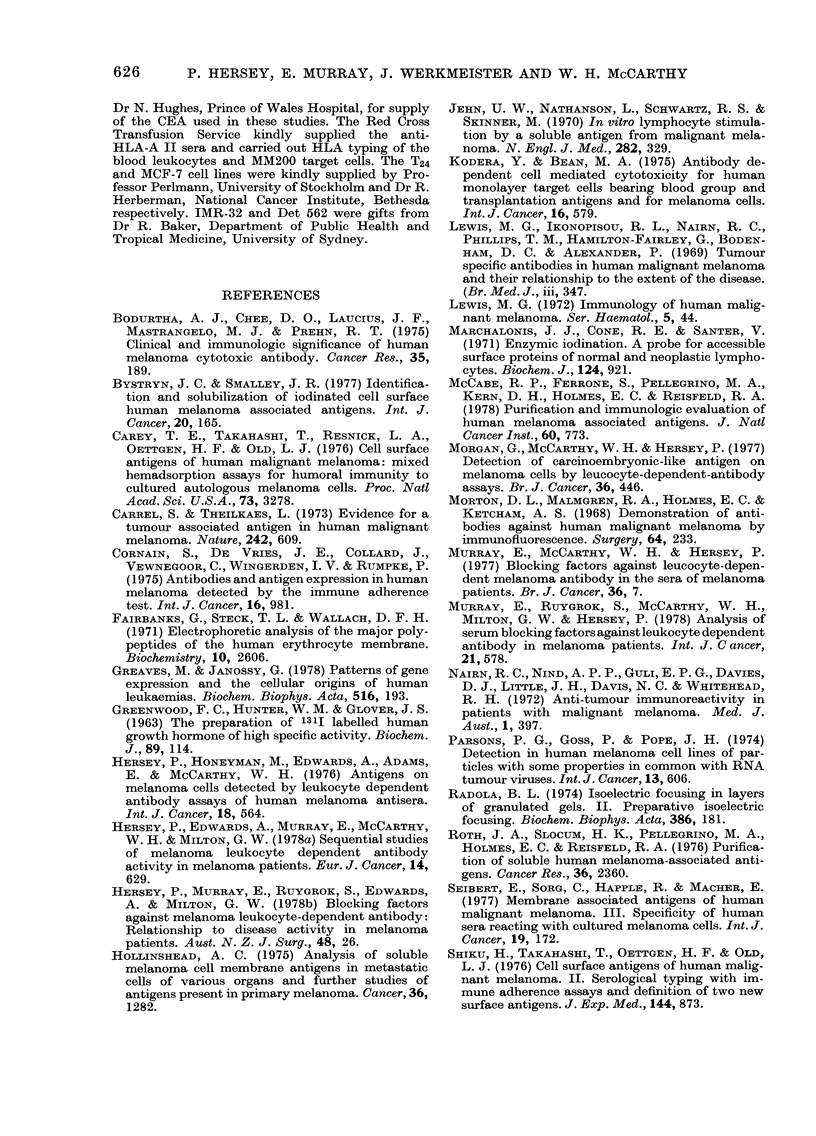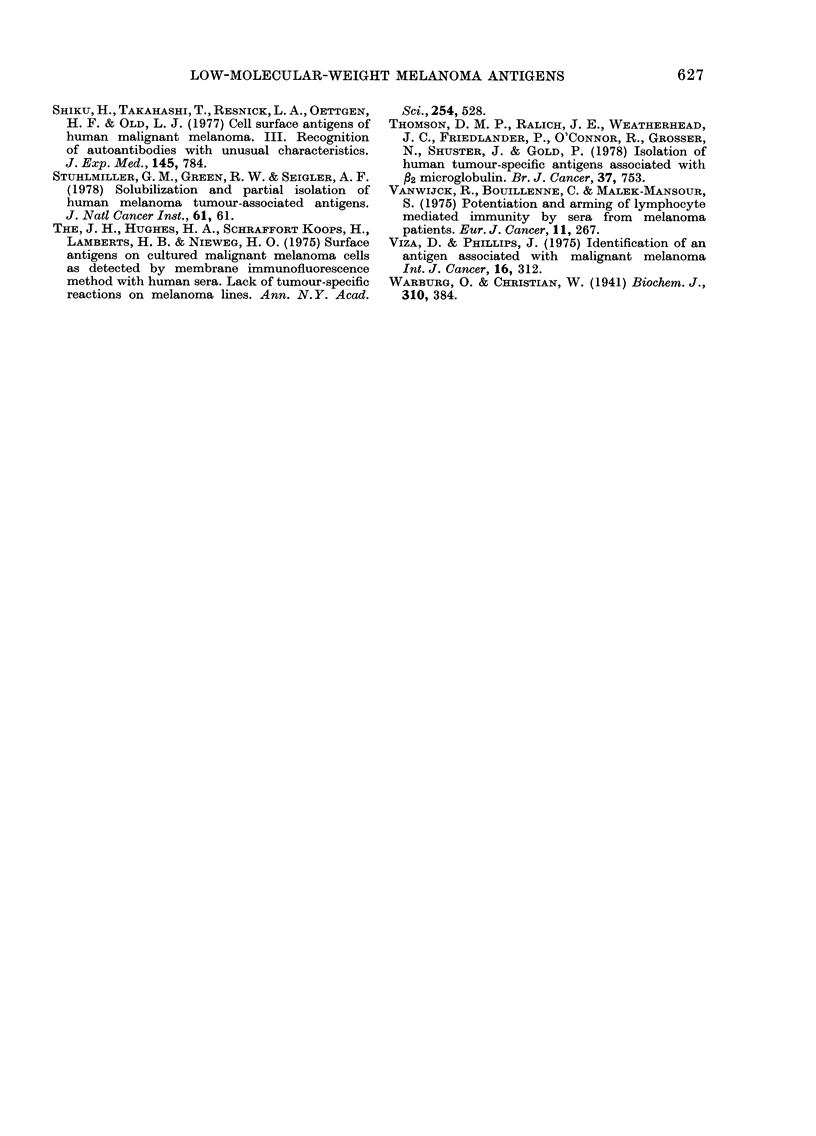# Detection of a low-molecular-weight antigen on melanoma cells by a human antiserum in leukocyte-dependent antibody assays.

**DOI:** 10.1038/bjc.1979.227

**Published:** 1979-10

**Authors:** P. Hersey, E. Murray, J. Werkmeister, W. H. McCarthy

## Abstract

Biochemical characterization of serologically detected human melanoma antigens was undertaken for the development of immunodiagnostic assays in melanoma. An antiserum from a human melanoma patient, which detected melanoma antigens expressed on a large proportion of different melanoma cells, was used in leucocyte-dependent cytotoxic antibody (LDA) 51Cr-release assays to monitor the purification of melanoma antigens in urea/acetate extracts of lactoperoxidase 125I-labelled melanoma cell membranes. The separation procedures included affinity chromatography on Concanavalin A, gel filtration on porous polyacrylamide beads and preparative isoelectric focusing. The fractions were also monitored by polyacrylamide electrophoresis in sodium dodecyl sulphate and by measurement of beta 2 microglobulin and carcinoembryonic antigen content. The antigens detected by this antiserum appeared to be acidic (pI 3.5) low-mol.-wt glycoproteins of approximately 15,000 daltons which were resistant to heating at 56 degrees C and digestion with neuraminidase, but susceptible to repeated freeze-thawing and trypsin digestion. They did not appear to be related to HLA antigens, beta 2 microglobulin or known foetal antigens. The nature of the antigens detected in these studies is as yet unknown, but they appear similar to those described in the sera and urine of melanoma patients in previous reports. Thes combined results and the frequent expression of these antigens on melanoma cells from different patients suggest that assays to detect this antigen may provide a valuable immunodiagnostic aid in the management of melanoma.


*Br. J. Cancer* (1979) **40**, 615

### DETECTION OF A LOW-MOLECULAR-WEIGHT ANTIGEN ON MELANOMA CELLS BY A HUMAN ANTISERUM IN LEUKOCYTE-DEPENDENT ANTIBODY ASSAYS

P. HERSEY, E. MURRAY, J. WERKMEISTER AND W. H. McCARTHY*

*From the Kanematsu Memorial Institute, Sydney Hospital, and *Melanoma Unit, Department of Surgery, University of Sydney, Sydney Hospital, Australia*

Received 15 May 1979 Accepted 8 June 1979

**Summary.—Biochemical characterization of serologically detected human melanoma antigens was undertaken for the development of immunodiagnostic assays in melanoma. An antiserum from a human melanoma patient, which detected melanoma antigens expressed on a large proportion of different melanoma cells, was used in leucocyte-dependent cytotoxic antibody (LDA) $^{51}$Cr-release assays to monitor the purification of melanoma antigens in urea/acetate extracts of lactoperoxidase $^{125}$I-labelled melanoma cell membranes. The separation procedures included affinity chromatography on Concanavalin A, gel filtration on porous polyacrylamide beads and preparative isoelectric focusing. The fractions were also monitored by polyacrylamide electrophoresis in sodium dodecyl sulphate and by measurement of $\beta_2$ microglobulin and carcinoembryonic antigen content.**

**The antigens detected by this antiserum appeared to be acidic (pI 3·5) low-mol.-wt glycoproteins of ~15,000 daltons which were resistant to heating at 56°C and digestion with neuraminidase, but susceptible to repeated freeze–thawing and trypsin digestion. They did not appear to be related to HLA antigens, $\beta_2$ microglobulin or known foetal antigens. The nature of the antigens detected in these studies is as yet unknown, but they appear similar to those described in the sera and urine of melanoma patients in previous reports. These combined results and the frequent expression of these antigens on melanoma cells from different patients suggest that assays to detect this antigen may provide a valuable immunodiagnostic aid in the management of melanoma.**

ANTIBODIES to antigens on melanoma cells have been detected in the sera of melanoma patients by a variety of methods such as immunofluorescence (Morton *et al.*, 1968; Lewis *et al.*, 1969; Lewis, 1972; Nairn, 1972; The *et al.*, 1975), complement-mediated cytotoxicity (Bodurtha *et al.*, 1975) immune adherence (Seibert *et al.*, 1977; Cornain *et al.*, 1975; Shiku *et al.*, 1976, 1977) and leucocyte-dependent cytotoxic antibody (LDA) assays (Hersey *et al.*, 1976, 1978*a*; Kodera & Bean, 1975; Vanwijck *et al.*, 1975).

However, the nature of the antigens detected by the human antisera in these studies is unknown. In some instances the antigens appeared specific for each individual (Lewis *et al.*, 1969; Lewis, 1972; Bodurtha *et al.*, 1975; Shiku *et al.*, 1976, 1977) whereas, in other studies, several different antigens showing partial cross-reactivity between melanoma cells from different patients were detected (Hersey *et al.*, 1976; Seibert *et al.*, 1977; Carey *et al.*, 1976; Shiku *et al.*, 1976, 1977).

The aim of this study was to define the biochemical nature of some of the antigens detected by human antisera on melanoma cells in the belief that this information might provide some understanding of their

Correspondence to: Dr P. Hersey, Medical Research Department, Kanematsu Memorial Institute, Sydney Hospital, Macquarie Street, Sydney, N.S.W. 2000, Australia.

biological nature and assist in the development of immunodiagnostic assays. The antigen selected for study was that detected by a human antiserum which showed partial cross-reactivity with a large proportion of melanoma cells. Our results indicate that the antigens detected by this antiserum appeared to be low-mol.-wt acidic glycoproteins of 15,000 daltons which were separable from HLA and foetal antigens on the cell surface. They appeared similar to those previously detected in sera and urine of melanoma patients (Murray *et al.*, 1978) and we consider that assays to detect this antigen may provide a useful immunodiagnostic aid in melanoma.

### MATERIALS AND METHODS

*Antisera.*—The serum Ch used to characterize the melanoma antigen in these studies was obtained from a 66-year-old woman with a long history of multiple local recurrences in her leg. These dated from shortly after removal of the primary melanoma 4 years previously, and her leg was eventually amputated 3 years after removal of the primary to control the local recurrences. The serum was obtained just before the clinical detection of disseminated melanoma in her liver.

Antisera to $\beta_2$ microglobulin ($\beta_2$M) and carcinoembryonic antigen (CEA) were from Dako (Copenhagen) and were used after absorption on human RBC and pooled human platelets respectively. Serum AE from a normal multiparous woman and JT from a female melanoma patient have been described previously (Hersey *et al.*, 1976). Sera Tit. and HI were from male melanoma patients and Jay from a female melanoma patient. They reacted with the MM200 line (see below) but not with cells from the non-melanoma cell lines used in this study. Sera Har and Joy were from patients with carcinoma of bladder and breast respectively. They reacted with the corresponding cell lines used in this study, but not with the other cell lines, including the melanoma cell lines. Mac was a serum from a patient with colon carcinoma, and was kindly supplied by Dr Nind of the Department of Pathology and Immunology, Monash University.

*Melanoma LDA assays and measurement of factors blocking melanoma LDA.*—The procedures involved in detection of melanoma LDA by $^{51}$Cr-release assays, and the use of these assays to measure blocking factors against the melanoma LDA, have been fully described in several recent publications (Murray *et al.*, 1977, 1978; Hersey *et al.*, 1978*a*).

In brief, 50 $\mu$l of the sensitizing antiserum at an appropriate dilution and 50 $\mu$l of the cell-membrane fraction in saline in 10-fold dilutions were added to $12 \times 75$mm round-bottomed plastic tubes (Filtrona, Melbourne). $3 \times 10^3$ $^{51}$Cr-labelled target cells in 200 $\mu$l of RPMI + 10% foetal bovine serum (FBS) and $3 \times 10^5$ effector cells in 200 $\mu$l RPMI + 10% FBS were added, and incubated overnight at 37°C. Effector cells were obtained by centrifugation of defibrinated venous blood from normal laboratory volunteers on Hypaque-Ficoll mixtures.

In all assays the samples being tested for LDA blocking were also added to cultures without sensitizing LDA to determine the effect, if any, on the natural killing activity of the effector cells. The level of natural killing in the presence of the test samples was taken as the baseline for measurement of LDA activity. Blocking of LDA activity was estimated by comparison of the per cent $^{51}$Cr release due to LDA in the absence and presence of the sample being tested for LDA blocking. The titre of LDA blocking was taken as the last dilution giving a 5% reduction in $^{51}$Cr release. This level of $^{51}$Cr release was more than 2 standard deviations from the points concerned, and was taken as the minimum definite evidence for LDA blocking.

*Melanoma cells.*—These were principally from the MM200 line established in culture from a primary melanoma in the Queensland Institute of Medical Research (Parsons *et al.*, 1974). Previous studies showed these cells had surface antigens detected by a high proportion of antisera from melanoma patients (Hersey *et al.*, 1976). Foetal antigens of undefined nature, CEA-like antigens and $\beta_2$ microglobulin have been detected on these cells (Hersey *et al.*, 1976; Morgan *et al.*, 1977). The cells were grown in monolayers in RPMI 1640 supplemented with 20% FBS (Australian Laboratory Services, Batch 64).

*Radioiodination of cell-surface macromolecules.*—Radiolabelling of the melanoma cell-surface molecules was carried out by lactoperoxidase iodination procedures modified from those described by Marchalonis *et al.* (1971). MM200 cells were harvested after a short exposure (5–10 min) to 0·25% trypsin. They were then incubated at 37°C in RPMI for 3–4 h to allow recovery of any surface antigens removed by the trypsin. Aliquots of $4 \times 10^6$ cells were then washed $3\times$ in Hanks' balanced salt solution (HBSS) and the cell pellets resuspended in 100 $\mu$l of phosphate buffered saline (PBS) pH 7·3, containing lactoperoxidase (Calbiochem B grade) at 500 $\mu$g/ml. To this was added 3 $\mu$l of $Na^{125}I$ (Amersham, Bucks, England) and 10 $\mu$l of $H_2O_2$ (3 mg/100 ml in PBS). The mixture was agitated, incubated for 5 min at 23°C and the addition of lactoperoxidase and $H_2O_2$ repeated. The reaction was stopped after 5 min by addition of cold PBS, and the cells were then washed twice in 25 ml of PBS.

*Extraction of melanoma surface antigens by urea/acetate.*—The radiolabelled cells were resuspended in 1 ml of a mixture of 10M urea in 1·5M acetic acid (pH 4·8) as described by Marchalonis *et al.* (1971) and incubated at 37°C for 2 h. The cells were then centrifuged at 400 *g* for 15 min and the supernatant containing the $^{125}I$-labelled proteins recovered and dialysed twice against 1 l of PBS.

*Affinity chromatography on Concanavalin A (Con A).*—The cell-membrane extract was applied to Con A in a column $0·9 \times 15$ cm and a graded elution procedure with $\alpha$-methyl glucopyranoside ($\alpha$MG) was used as described fully in a previous report (Murray *et al.*, 1978).

*Gel exclusion chromatography.*—This was carried out on Biogel $P_{100}$, 100–200 mesh and on Biogel $P_{30}$, 100–200 mesh (Bio-Rad Laboratories) in $1·5 \times 90$cm columns (k 15/90 Pharmacia). The gel was prepared according to manufacturer's instructions by hydration in 0·1M EDTA in PBS (pH 5·2) at 90°C for 4 h. The membrane extract was eluted in 0·1M EDTA in PBS at a flow rate of 20 ml/h and 100 2ml samples were collected. Radioactivity and UV absorbance of the fractions at 260 and 280 nm were measured, and the fractions were then pooled on the basis of the radioactivity counts. The pooled fractions were concentrated back to 2 ml in an Amicon Diaflo cell using UM2 membranes. They were then dialysed twice against 1 l of PBS.

The column was calibrated with bovine serum albumin, 68,000 dalton (Sigma) ovalbumin, 43,000 daltons (Sigma) cytochrome C, 12,000 daltons (Sigma) and blue dextran 2000 (Pharmacia Fine Chemicals).

*Polyacrylamide electrophoresis in sodium dodecyl sulphate (PAGE-SDS).*—The method described by Fairbanks *et al.* (1971) was used as described previously (Murray *et al.*, 1978) using $100 \times 6$mm glass tubes and gels made from 7·5% acrylamide and 0·3% methylene bis acrylamide. Gels were sliced mechanically into 2mm sections and counted in a gamma counter. Mobility of radiolabelled peaks was expressed relative to bromophenol blue.

*Radiolabelling of melanoma antigen fractions.*—Fractions from gel filtration were labelled with $^{125}I$ by the chloramine T method of Greenwood *et al.* (1963). 10–20 $\mu$g of the fractions in 20 $\mu$l were added to 1 mCi of $Na^{125}I$ (Amersham, Bucks, England) and 10 $\mu$l of chloramine T (2 mg/ml) for 60 s, then 20 $\mu$l of sodium metabisulphate (7·2 mg/ml) was added to reduce unbound iodide. Free $^{125}I$ was removed by dialysis twice against 1 l of 0·8% NaCl in 24 h.

*Digestion of melanoma antigen fractions with trypsin and neuraminidase.*—Tryspin digestion. 50 $\mu$l of the antigen fraction was incubated with 50 $\mu$l of 0·25% Trypsin (Difco) in HBSS at 37°C for 60 min. 50 $\mu$l of FBS was then added and the mixture used directly in the melanoma LDA assays.

Neuraminidase digestion was carried out using neuraminidase insolubilized on agarose (Sigma N 5254) 0·03 unit (150 $\mu$l) was added to 400 $\mu$l of the antigen fraction and incubated with mixing at 37°C for 30 min. The supernatant was then recovered after centrifugation and tested for inhibitory activity against melanoma LDA.

*Estimation of protein concentration.*—This was estimated by measurement of the UV absorption of the sample at 260 and 280 nm as described by Warburg & Christian (1941). Insufficient material was available for protein estimations by the Lowry method.

*Preparative isoelectricfocusing.*—This was carried out on a flat bed (Radola, 1974) in Ultrodex (LKB) using LKB model 2117 Multiphor equipment. In initial experiments 1 ml of the antigen fraction from the Biogel $P_{100}$ or $P_{30}$ gel filtration was mixed with 2·5 ml of ampholine, pH range 3–9, and 46·5 ml of distilled water. 2 g of ultrodex was added to this and the mixture placed on a flat glass plate 30 cm by 3 cm. After nearly complete evaporation a constant power of 2 W was applied for 18 h. At this time a grid with 30

spaces was applied, the pH of each section measured and the antigen recovered from each slice by several saline washes of the ultrodex slices. After dialysis these were concentrated on UM2 membranes in a 10 ml Amicon cell and tested for melanoma LDA blocking.

In subsequent studies 1 ml of the antigen solution was run with ampholines over the pH range 2–4, using the above conditions.

### RESULTS

### *Specificity of melanoma antiserum Ch*

The antiserum Ch was tested against a number of cultured non-melanoma target cells, cultured melanoma cells and primary cultures of melanoma cells established from melanomas removed at surgery. The results of these assays are shown in Table I in terms of LDA titres. As can be seen there was no reaction with a large variety of cultured non-melanoma target cells, all of which showed susceptibility to killing in LDA assays with appropriate antisera. Assays against several different freshly isolated carcinomas (breast (3) ovarian (1) colon (1) and basal cell carcinoma (3)) were also negative.

The reaction against 1 of the 3 freshly isolated breast carcinoma cells was weak and seen only at a dilution of 1 in 10. Repeat assays against this particular target cell were not possible with antisera absorbed on foetal tissue, but previous studies have shown that weak cross-reactivity of this nature was removed after absorption of the antisera on foetal tissue. (Hersey *et al.*, 1976.)

There was reactivity with ~60% of the 26 primary cultures of melanoma cells, which was consistent with the restricted cross-reactivity of human melanoma antisera noted in previous studies (Hersey *et al.*, 1976).

The specificity of the serum was also assessed by absorption on $\frac{1}{3}$ its volume of foetal brain from a 16–20-week foetus, foetal thymus and spleen from a 16–18-week foetus and melanocytes from the uveal tract of a freshly excised eye, for 30 min at 37°C and 1 h at 4°C. No reduction in titre was noted after these absorptions. The serum was also passed over $\beta_2$ microglobulin ($\beta_2$M) coupled to Sepharose 4B and carcinoembryonic antigen (CEA) (kindly supplied by Dr N. Hughes, Prince of Wales Hospital, N.S.W.) bound to Sepharose 4B. No reduction in titre was found, whereas passage of antisera against $\beta_2$M and CEA completely removed the activity from equivalent amounts of sera.

### *Analysis of melanoma cell-membrane extracts by affinity chromatography on Con A*

A dialysed urea/acetate cell-membrane

TABLE I.—*Specificity of melanoma serum Ch in LDA assays*

| Reaction against non-melanoma target cells | | Reaction against melanoma target cells | | | | | |
|---|---|---|---|---|---|---|---|
| Target cell | LDA titre | Target cell | LDA titre | Target cell | LDA titre | Target cell | LDA titre |
| Chang | 0 | *MM200 | $10^{-3}$ | Hos | $10^{-1}$ | Tod | $10^{-3}$ |
| †IMR32 | 0 | MM96 | $10^{-1}$ | Nug | $10^{-3}$ | Sho | 0 |
| HT29 | 0 | MM127 | 0 | Bur | $10^{-3}$ | Fle | $10^{-3}$ |
| T24 | 0 | MM170 | $10^{-2}$ | Bro | 0 | Hef | 0 |
| Det562 | 0 | Odm | $10^{-2}$ | A33 | 0 | Pug | $10^{-2}$ |
| MCF-7 | 0 | Jem | 0 | Ikn | $10^{-3}$ | Hog | $10^{-2}$ |
| $B_1$ | $10^{-1}$ | Mar | $10^{-2}$ | Woo | $10^{-3}$ | Cal | $10^{-3}$ |
| PHA-Ly(4) | 0 | Sim | 0 | Sims | $10^{-1}$ | Kel | $10^{-3}$ |
| HL60 | 0 | Pet | $10^{-3}$ | Law | $10^{-1}$ | Bor | 0 |

* MM = Melanoma cell lines. Letters refer to individual patients.

† IMR32 = neuroblastoma, HT29 = colon carcinoma, T24 = bladder carcinoma, Det562 = carcinoma of pharynx, MCF-7 = breast carcinoma, HL60 = acute myeloid leukaemia, $B_1$ = freshly isolated breast carcinoma, PHA-Ly = phytohaemagglutinin transformed blood lymphocytes whose HLA phenotypes were as follows: A2, 3, B7; A1, 2, B7,15; A24,31/30. B7,27; A2,11, B27,41.

FIG. 1.—Melanoma LDA blocking by fractions from Con A affinity chromatography of melanoma cell-membrane extract. Ex=urea/acetate extract of MM200 cells. 0 = fraction not binding to Con A, and 5, 50 and 100 indicate fractions eluted at these concentrations (mg/100 ml) of α-methyl glucopyranoside (αMG). Blocking of melanoma LDA is shown by original extract and fractions eluted with 100 mg and 50 mg of αMG. T = MM200 target cells alone. +E = MM200 + 3 × $10^5$ effector cells from normal subject. +ab = MM200 + effector cells + melanoma Ab, Ch, at a final dilution of 1:200. S.e. < 2% ●, with and ○, without antigen.

extract from 50 × $10^6$ MM200 cells in 9 ml was applied to 150 ml of Con A Sepharose and eluted from the Con A with 0·5M NaCl in HBSS containing 0, 5, 50 and 100 mg of αMG. These fractions and the original extract were tested for their melanoma LDA-neutralizing activity as shown in Fig. 1. Blocking was seen to a titre of at least $10^{-3}$ in the fraction eluted with 100 mg of αMG and $10^{-2}$ in the 50mg αMG eluate. It may also be noted that stimulation of cytotoxicity by the cells used as antibody-dependent effector cells was noted against the target cells in all the fractions eluted with αMG. In some studies LDA blocking was seen in the non-binding fraction (Con $A^-$) but this was removed by a second passage over the Con A, and presumably was due to overloading the column. The above results were consistent in 4 experiments. The $^{125}I$ counts eluted with 100 mg αMG were 8·6, 14·5, 5·2 and 11% of the total counts eluted in 4 experiments.

*Gel chromatography of melanoma antigen fraction from Con A affinity chromatography*

The elution profile of radiolabelled

FIG. 2.—"BioGel $P_{100}$" filtration profile of the 100mg αMG eluate from Con A affinity chromatography of the $^{125}I$-labelled melanoma extract. E = excluded fraction, BSA, Ov, Ch and CC indicate elution position of BSA (68,000 daltons), Ovalbumin (43,000 daltons), Chymotrypsinogen (25,700 daltons) and Cytochrome C (12,000 daltons). The 100 mg αMG eluate fractions were pooled into A, B, $B_1$, C, D and $D_1$ and E as indicated. Mol. wts of B, C, $D_1$ and E were ~60,000, 35,000, 15,000 and 7,500 respectively.

FIG. 3.—LDA blocking by the gel-filtration fractions indicated in Fig. 2 against melanoma serum Ch. Symbols as for Fig. 1. LDA blocking was most pronounced in the low-mol.-wt fractions D and $D_1$. S.e. < 2%.

material from the BioGel $P_{100}$ column of the 100mg αMG eluate is shown in Fig. 2. Major radiolabelled peaks were eluted before and after that of cytochrome C (12,000 daltons) with mol. wt of ~15,000 and 7500. Smaller peaks were eluted between ovalbumin and chymotrypsin of ~35,000 and close to BSA of ~68,000. A small peak was present in the exclusion volume. The 100mg αMG fractions from gel filtration were pooled as indicated in the figure, into fractions referred to as A,B,$B_1$,C,D,$D_1$ and E. They were then dialysed, concentrated back to the original volume, applied to the column (8 ml) and tested for blocking against melanoma LDA of Ch, as shown in Fig. 3.

The low-mol.-wt Fractions D and $D_1$ of the 100mg αMG eluate had the most pronounced blocking activity (titre greater than $10^{-3}$) but weak blocking was also seen in Fractions E and B. These results were repeated on 4 occasions, and on each occasion the major LDA blocking activity was in the D fraction eluting before cytochrome C. The main variations between experiments were (1) the excluded Fraction A had relatively more radiolabelled counts in some experiments, (2) the LDA blocking activity in Fractions A, B and C varied from 0 to $10^{-1}$.

*Further gel chromatography of the low-mol.-wt fraction of melanoma cell-membrane extract*

To more closely define the low-mol.-wt fraction $D_1$, 7 ml of this fraction was applied to a Biogel $P_{30}$ column, which separated the material as shown in Fig. 4. Peaks of radioactivity were seen at 15,000 and 30,000 and a small peak in the excluded fraction. The fractions were re-combined into Fractions A, B, C and D as indicated, dialysed, concentrated on UM2 membranes and tested in LDA assays as shown in Fig 5. This assay was carried out simultaneously with that shown in Fig. 3. The major blocking activity was again in the 15,000 fraction (titre $>10^{-3}$) but small amounts of LDA blocking were seen in all fractions.

*Characterization of melanoma cell-membrane fractions by PAGE-SDS*

In parallel with the separation procedures and characterization by LDA blocking described above the fractions were analysed by PAGE-SDS. Representative results of these studies on fractions obtained by gel filtration on Biogel $P_{30}$ are shown in Fig. 6. A, B, C and D refer to the fractions shown in Fig. 4. All frac-

FIG. 4.—Gel filtration profile of the low-mol.-wt fraction $D_1$ from Biogel $P_{100}$ on $P_{30}$. Radiolabelled peaks were seen predominantly at 15,000 and 30,000 daltons. The fractions were recombined into fractions A, B, C, and D, as shown.

FIG. 5.—LDA blocking by gel-filtration fractions from BioGel $P_{100}$. Blocking was again predominantly in low-mol.-wt fraction D. Symbols as for Fig. 1.

tions were relabelled with $^{125}$I by the chloramine T method.

In all instances in which the fraction had LDA blocking activity, a low-mol.-wt fraction of 15,000 was detected in PAGE-SDS with relative mobility of 0·85 migrating close to cytochrome C (relative mobility 0·925). This is most clearly seen in Fig. 6D. Fractions without LDA blocking activity did not have peaks with this mobility. A lower-mol.-wt factor was usually detected with similar mobility to cytochrome C, but this did not correlate with LDA blocking. Similarly, fractions with mobility close to 0·7 (25–30,000) and 0·45 (~60,000) were evident but showed a variable correlation with LDA blocking.

*Specificity of LDA blocking activity of the melanoma antigen fraction*

The low-mol.-wt Fraction D from the Biogel $P_{100}$ fraction was tested in LDA assays against MM200 using a variety of melanoma and non-melanoma antisera. The results in Table II indicate that LDA activity of anti $\beta_2$M and CEA and anti-HLA-A 11 against MM200 target cells was not inhibited. This also applied to the antiserum AE from a normal subject, which, as previously shown, appeared to be directed to foetal antigens (Hersey *et al.*, 1976). The fractions did, however, inhibit the LDA activity of a number of antisera from melanoma patients as shown in the table. The specificity of the fraction was further assessed by addition to LDA assays against a number of non-melanoma

FIG. 6.—PAGE-SDS profiles of the melanoma cell-membrane fractions obtained by gel filtration of $P_{100}$ $D_1$ fraction of BioGel $P_{30}$. BSA, Ov, Ch and CC as in Fig. 2.

See text for description and Fig. 5 for LDA blocking activity of the corresponding fractions.

FIG. 7.—Characterization of the BioGel $P_{30}$ "D" antigen fraction by isoelectric focusing in Ultrodex, using ampholines over the pH range 2–4. LDA blocking was predominantly focused at pH 3·5 and pH 2·5. CMC = cell-mediated cytotoxicity.

target cells sensitized with LDA specific to the target cell. These are also indicated in Table II. The antisera against the carcinoma cell lines were from human patients with the corresponding carcinomas. They all had titres of $10^2$–$10^3$. The anti-HLA-A II antiserum was from a male patient bearing a renal homograft and had a titre of $10^{-2}$. The melanoma antigen fraction did not block the LDA activity of these sera.

*LDA blocking by antigen extract from bladder-carcinoma cell line*

As a further check of the specificity of the antigen fraction, $50 \times 10^6$ $T_{24}$ bladder carcinoma cells were extracted with urea/acetate and processed to the Biogel $P_{100}$ stage. A low-mol.-wt fraction equivalent to the D fraction seen in the Biogel $P_{100}$ profile of the melanoma extracts was not seen in the bladder-carcinoma extracts. The Con A 100mg αMG eluate of the bladder carcinoma extract was therefore used to test if this fraction contained material which would block the antiserum Ch in LDA assays against the MM200 target cell. As shown in Table II, no blocking activity was detected against this antiserum, but it did block the LDA activity of an antiserum from a bladder-

TABLE II.—*Specificity of LDA blocking by melanoma antigen fraction*

| Target cell | Sensitizing antisera | LDA blocking titre | Target cell | Sensitizing antisera | LDA blocking titre |
|---|---|---|---|---|---|
| MM200 | Anti $\beta_2$M | 0 | Chang | Rabbit anti-Chang | 0 |
| ,, | Anti CEA | 0 | T24 | Har‡ | 0 |
| ,, | | | T24† | Har‡ | $10^3$ |
| ,, | JT* | $10^2$ | HT29 | Mac‡ | 0 |
| ,, | Jay* | 0 | MCF-7 | Joy‡ | 0 |
| ,, | Tit* | $10^3$ | PHA-Lym | Anti-HLA-A II | 0 |
| ,, | AE | 0 | | | |
| ,, | Ch* | $10^3$ | | | |
| ,, | Ch† | 0 | | | |
| ,, | Anti-HLA-A II | 0 | | | |

See Table I for Codes to cell lines used.
* Antisera from melanoma patients.
† Addition of antigen from T24 bladder-carcinoma cells.
‡ Antisera from patients with carcinoma corresponding to origin of cell line.
Target cells were sensitized with the lowest dilution of the antiserum giving maximal cytotoxicity against target cells.

carcinoma patient against the $T_{24}$ bladder-carcinoma cell line.

*Assays of melanoma antigen extracts for $\beta_2M$ and CEA*

The urea/acetate extract of $50 \times 10^6$ MM200 cells, and the Con A 100mg αMG eluate were assayed by commercial radioimmunoassays for $\beta_2$M and CEA. A CEA concentration of 4·2 ng/ml and a $\beta_2$M concentration of 10 μg/ml were detected in the original extract. Less than 0·4 ng/ml of CEA was detected in the 100mg αMG eluate. No $\beta_2$M was detectable in the Biogel $P_{100}$ fractions.

*Stability to physical and enzymatic agents*

The low-mol.-wt fraction D from the Biogel $P_{100}$ gel filtration procedure was subjected to freeze-thawing ×3, heating to 56°C and 95°C for 30 min and digestion with Trypsin and Neuraminidase as described. The treated and untreated fractions were then tested for LDA blocking in assays of serum Ch against the MM200 target cell. The results indicated that the antigen fraction was resistant to heating to 56°C for 30 min and digestion with neuraminidase, but was susceptible to freeze-thawing, heating to 95°C and digestion with trypsin. Similar results were obtained in 2 experiments with successive D fractions from the BioGel 100 filtration procedure.

*Analysis of the low-mol.-wt fraction by isoelectric focusing*

In preliminary experiments using ampholines over the range pH 3–9 the LDA blocking activity of the low-mol.-wt fraction D from the BioGel $P_{30}$ column was found to focus at the pH 3·5–3·8 end of the bed. The BioGel $P_{30}$ fraction was therefore run subsequently in ampholines over the pH range 2–4. The results shown in Fig. 7 indicate that the LDA blocking activity localized mainly at the pH range 3·45–3·55, but that smaller amounts were also detected at the pH range 2·45–2·55. Stimulation of cytotoxicity by the effector cells was noted at the pH range 2·3–2·4 and 3·3–3·6. When the LDA blocking fraction localizing at pH 3·5 was re-run on PAGE-SDS, radiolabelled peaks were noted at 15, 30, 45 and 60,000 daltons. Percentage recovery of $^{125}$I counts approached 90%. The fraction with LDA blocking activity localizing at about pH 3·5 in this study accounted for 22% of the counts recovered.

### DISCUSSION

The nature of the antigen detected by this antiserum is unknown. It appeared to be expressed on a high proportion (60%) of melanoma cells taken from surgical specimens, and was presumably similar to antigens showing partial cross-reactivity between different melanoma cells described in previous studies (Hersey *et al.*, 1976; Shiku *et al.*, 1976; Seibert *et al.*, 1977). The antiserum did not react with a large variety of cell lines established from several different carcinomas or other malignancies. Tests against PHA-transformed lymphocytes expressing a variety of HLA antigens were also negative. These results suggest the antiserum was not detecting HLA antigens, antigens associated with tissue cultures or foetal antigens common to carcinomas, lymphoid or haemopoietic malignancies. Absorption on foetal brain, thymus and spleen cells or CEA bound to sepharose did not cause loss of activity, which also argued against the specificity of the antiserum being directed to foetal antigens.

Greaves & Janossy (1978) reported that some antigens thought to be tumour-associated antigens on acute lymphatic leukaemia cells were instead differentiation antigens. Absorption of the antiserum used in this study on melanocytes from the uveal tract of a freshly excised eye of a foetus or adult, did not remove significant amounts of LDA activity against melanoma cells. This result argued against the antigen detected being a melanocyte differentiation antigen, but we cannot exclude that it may be expressed by melanocytes at different stages of differentiation from that used in these studies.

The use of LDA assays to define antigen could be questioned, in that it is possible for the effector cells in the assay to be inhibited non-specifically by toxic components in the membrane fractions. This was one of the reasons for the use of urea/acetate for the extraction procedure, since it was easier to remove than detergents such as NP40 and Triton X100. In preliminary experiments, the titre of LDA blocking activity obtained by urea/acetate was found comparable to that of NP40 and Triton X100.

The specificity of the LDA blocking for melanoma antigens was shown by addition of the fraction to assays of various sera against the MM200 target cell and a variety of non-melanoma target cells. The results indicated that it was only the melanoma antisera that were inhibited by the melanoma antigen fraction. In particular, antisera to HLA antigens and $\beta_2$M, and a human antiserum to foetal antigens on the melanoma cell, were not inhibited by the antigen fraction. Conversely, an antigen fraction from cultured bladder-carcinoma cells did not inhibit melanoma LDA, but did inhibit LDA to bladder-carcinoma cells. The results indicated that the blocking seen was not due to non-specific blocking of the effector cell but was specific for the antigen detected by the antiserum in the assays.

The methods used in these studies were adapted from those used previously to characterize a factor in the sera of melanoma patients which blocked melanoma LDA activity (Murray *et al.*, 1977; 1978). Affinity chromatography on Con A proved to be the most useful single procedure in purification of the antigen, and also provided evidence that it was a mannose- and/or glucose- containing glycoprotein. The subsequent gel filtration steps on polyacrylamide beads indicated that the antigen-containing fractions were ~15,000 mol. wt. It should be noted that the results of gel filtration with polyacrylamide beads were highly reproducible, but were less so with Sephadex G50 or G100. This may have indicated interaction of the material with the Sephadex. Fractions of ~15,000 mol.-wt were also seen in the PAGE-SDS studies and could be correlated with the LDA blocking activity of these fractions. PAGE-SDS studies of the 15,000 mol.-wt fraction consistently showed peaks at 30,000 and 60,000, which suggested that the antigenic material may have aggregated despite the use of SDS. Similar explanations may account for the localization of LDA blocking activity at pH 3·5 and pH 2·5 by isoelectric focusing, or this may represent two different antigens.

The antigenic activity appeared to be lost after digestion with trypsin or freeze-thawing, which suggested that the protein component formed part of the antigenic determinants of the molecule. There was no loss of activity after digestion with neuraminidase, which indicated that terminal sialic acid did not form part of the antigenic determinants. A more comprehensive study using different glycosidases is, however, required before the role of the glycoside component in the antigenicity of the molecules can be assessed.

There is now considerable diversity of opinion in the literature on the biochemical nature of melanoma antigens, which to a large degree may be a reflection of the different methods used to characterize the antigens. Bystryn & Smalley (1977) using double antibody precipitation methods, indicated that the mol. wt of the antigens was greater than 100,000. Similar results had previously been obtained by Viza & Phillips (1975) using crossover electrophoresis to detect the melanoma antigen. Both studies used antisera raised in rabbits against the antigen preparations, and it is possible that the difference noted in our studies may reflect the recognition of different antigens on melanoma cells by rabbits compared to humans.

Melanoma antigens producing delayed skin-test responses were shown to be glycolipoproteins by Hollinshead (1975) and both high and low-mol.-wt proteins by Roth *et al.* (1976). Jehn *et al.* (1970) found melanoma antigens which induced lymphocyte transformation to be $\beta$ globulins of mol-wt $< 40{,}000$. Rather similar results were obtained by Carrel & Theilkaes (1973) in gel-diffusion assays.

Thomson *et al.* (1978) used leucocyte adherence inhibition assays to characterize papain extracts of melanoma cells, and found the active fractions could be resolved by gel filtration and PAGE-SDS into several fractions with mol. wts of $\sim 12$, 25 and 40,000. The low-mol.-wt fraction was thought to be $\beta_2$M and it was postulated that tumour-associated antigens might be associated with $\beta_2$M. The small 15,000 mol.-wt fraction in our studies was unlikely to be $\beta_2$M in that it was consistently larger than $\beta_2$M by gel filtration and PAGE-SDS. $\beta_2$M was not detected by commercial radioimmunoassays of the purified antigen fractions, which did not inhibit antisera to $\beta_2$M in LDA assays against the melanoma target cell. Our results also appear to exclude association of melanoma antigen with HLA antigens because the purified fractions did not inhibit HLA antisera against the MM200 target cell nor against blood leucocytes. These results are similar to those of McCabe *et al.* (1978) and Stuhlmiller *et al.* (1978).

The latter authors found that melanoma antigens could be detected in fractions with mol.-wts of 48,000, 25,000, 17,000 and 13,000 by inhibition of a chimpanzee antiserum to melanoma cells. All but the 13,000 fraction contained foetal antigens. Our results support these findings of melanoma antigens in low-mol.-wt fractions of cell membrane extracts.

The low-mol.-wt fraction defined in these studies is similar to that described in sera of melanoma patients in a previous report (Murray *et al.*, 1978). In preliminary studies it was also found that melanoma LDA blocking activity in sera was related to tumour growth (Hersey *et al.*, 1978*b*). We are therefore encouraged to believe that the antigen fraction defined in these studies is identical to that detected in the circulation and urine of melanoma patients, and, in view of the common expression of this antigen on melanoma cells, monitoring of this fraction might provide a valuable immunodiagnostic aid in management of melanoma. The biological nature of the antigens detected in this study and their importance in the tumour relationship remain to be studied.

This work was supported by the N.S.W. State Cancer Council, the National Health and Medical Research Council and the Clive & Vera Ramaciotti Foundation.

We wish to thank Mrs A. Edwards for helpful assistance in supply of cultured tumour cells and

Dr N. Hughes, Prince of Wales Hospital, for supply of the CEA used in these studies. The Red Cross Transfusion Service kindly supplied the anti-HLA-A II sera and carried out HLA typing of the blood leukocytes and MM200 target cells. The $T_{24}$ and MCF-7 cell lines were kindly supplied by Professor Perlmann, University of Stockholm and Dr R. Herberman, National Cancer Institute, Bethesda respectively. IMR-32 and Det 562 were gifts from Dr R. Baker, Department of Public Health and Tropical Medicine, University of Sydney.

### REFERENCES

BODURTHA, A. J., CHEE, D. O., LAUCIUS, J. F., MASTRANGELO, M. J. & PREHN, R. T. (1975) Clinical and immunologic significance of human melanoma cytotoxic antibody. *Cancer Res.*, **35**, 189.

BYSTRYN, J. C. & SMALLEY, J. R. (1977) Identification and solubilization of iodinated cell surface human melanoma associated antigens. *Int. J. Cancer*, **20**, 165.

CAREY, T. E., TAKAHASHI, T., RESNICK, L. A., OETTGEN, H. F. & OLD, L. J. (1976) Cell surface antigens of human malignant melanoma: mixed hemadsorption assays for humoral immunity to cultured autologous melanoma cells. *Proc. Natl Acad. Sci. U.S.A.*, **73**, 3278.

CARREL, S. & THEILKAES, L. (1973) Evidence for a tumour associated antigen in human malignant melanoma. *Nature*, **242**, 609.

CORNAIN, S., DE VRIES, J. E., COLLARD, J., VEWNEGOOR, C., WINGERDEN, I. V. & RUMPKE, P. (1975) Antibodies and antigen expression in human melanoma detected by the immune adherence test. *Int. J. Cancer*, **16**, 981.

FAIRBANKS, G., STECK, T. L. & WALLACH, D. F. H. (1971) Electrophoretic analysis of the major polypeptides of the human erythrocyte membrane. *Biochemistry*, **10**, 2606.

GREAVES, M. & JANOSSY, G. (1978) Patterns of gene expression and the cellular origins of human leukaemias. *Biochem. Biophys. Acta*, **516**, 193.

GREENWOOD, F. C., HUNTER, W. M. & GLOVER, J. S. (1963) The preparation of $^{131}I$ labelled human growth hormone of high specific activity. *Biochem. J.*, **89**, 114.

HERSEY, P., HONEYMAN, M., EDWARDS, A., ADAMS, E. & MCCARTHY, W. H. (1976) Antigens on melanoma cells detected by leukocyte dependent antibody assays of human melanoma antisera. *Int. J. Cancer*, **18**, 564.

HERSEY, P., EDWARDS, A., MURRAY, E., MCCARTHY, W. H. & MILTON, G. W. (1978*a*) Sequential studies of melanoma leukocyte dependent antibody activity in melanoma patients. *Eur. J. Cancer*, **14**, 629.

HERSEY, P., MURRAY, E., RUYGROK, S., EDWARDS, A. & MILTON, G. W. (1978b) Blocking factors against melanoma leukocyte-dependent antibody: Relationship to disease activity in melanoma patients. *Aust. N. Z. J. Surg.*, **48**, 26.

HOLLINSHEAD, A. C. (1975) Analysis of soluble melanoma cell membrane antigens in metastatic cells of various organs and further studies of antigens present in primary melanoma. *Cancer*, **36**, 1282.

JEHN, U. W., NATHANSON, L., SCHWARTZ, R. S. & SKINNER, M. (1970) *In vitro* lymphocyte stimulation by a soluble antigen from malignant melanoma. *N. Engl. J. Med.*, **282**, 329.

KODERA, Y. & BEAN, M. A. (1975) Antibody dependent cell mediated cytotoxicity for human monolayer target cells bearing blood group and transplantation antigens and for melanoma cells. *Int. J. Cancer*, **16**, 579.

LEWIS, M. G., IKONOPISOU, R. L., NAIRN, R. C., PHILLIPS, T. M., HAMILTON-FAIRLEY, G., BODENHAM, D. C. & ALEXANDER, P. (1969) Tumour specific antibodies in human malignant melanoma and their relationship to the extent of the disease. (*Br. Med. J.*, iii, 347.

LEWIS, M. G. (1972) Immunology of human malignant melanoma. *Ser. Haematol.*, **5**, 44.

MARCHALONIS, J. J., CONE, R. E. & SANTER, V. (1971) Enzymic iodination. A probe for accessible surface proteins of normal and neoplastic lymphocytes. *Biochem. J.*, **124**, 921.

MCCABE, R. P., FERRONE, S., PELLEGRINO, M. A., KERN, D. H., HOLMES, E. C. & REISFELD, R. A. (1978) Purification and immunologic evaluation of human melanoma associated antigens. *J. Natl Cancer Inst.*, **60**, 773.

MORGAN, G., MCCARTHY, W. H. & HERSEY, P. (1977) Detection of carcinoembryonic-like antigen on melanoma cells by leucocyte-dependent-antibody assays. *Br. J. Cancer*, **36**, 446.

MORTON, D. L., MALMGREN, R. A., HOLMES, E. C. & KETCHAM, A. S. (1968) Demonstration of antibodies against human malignant melanoma by immunofluorescence. *Surgery*, **64**, 233.

MURRAY, E., MCCARTHY, W. H. & HERSEY, P. (1977) Blocking factors against leucocyte-dependent melanoma antibody in the sera of melanoma patients. *Br. J. Cancer*, **36**, 7.

MURRAY, E., RUYGROK, S., MCCARTHY, W. H., MILTON, G. W. & HERSEY, P. (1978) Analysis of serum blocking factors against leukocyte dependent antibody in melanoma patients. *Int. J. C ancer*, **21**, 578.

NAIRN, R. C., NIND, A. P. P., GULI, E. P. G., DAVIES, D. J., LITTLE, J. H., DAVIS, N. C. & WHITEHEAD, R. H. (1972) Anti-tumour immunoreactivity in patients with malignant melanoma. *Med. J. Aust.*, **1**, 397.

PARSONS, P. G., GOSS, P. & POPE, J. H. (1974) Detection in human melanoma cell lines of particles with some properties in common with RNA tumour viruses. *Int. J. Cancer*, **13**, 606.

RADOLA, B. L. (1974) Isoelectric focusing in layers of granulated gels. II. Preparative isoelectric focusing. *Biochem. Biophys. Acta*, **386**, 181.

ROTH, J. A., SLOCUM, H. K., PELLEGRINO, M. A., HOLMES, E. C. & REISFELD, R. A. (1976) Purification of soluble human melanoma-associated antigens. *Cancer Res.*, **36**, 2360.

SEIBERT, E., SORG, C., HAPPLE, R. & MACHER, E. (1977) Membrane associated antigens of human malignant melanoma. III. Specificity of human sera reacting with cultured melanoma cells. *Int. J. Cancer*, **19**, 172.

SHIKU, H., TAKAHASHI, T., OETTGEN, H. F. & OLD, L. J. (1976) Cell surface antigens of human malignant melanoma. II. Serological typing with immune adherence assays and definition of two new surface antigens. *J. Exp. Med.*, **144**, 873.

Shiku, H., Takahashi, T., Resnick, L. A., Oettgen, H. F. & Old, L. J. (1977) Cell surface antigens of human malignant melanoma. III. Recognition of autoantibodies with unusual characteristics. *J. Exp. Med.*, **145**, 784.

Stuhlmiller, G. M., Green, R. W. & Seigler, A. F. (1978) Solubilization and partial isolation of human melanoma tumour-associated antigens. *J. Natl Cancer Inst.*, **61**, 61.

The, J. H., Hughes, H. A., Schraffort Koops, H., Lamberts, H. B. & Nieweg, H. O. (1975) Surface antigens on cultured malignant melanoma cells as detected by membrane immunofluorescence method with human sera. Lack of tumour-specific reactions on melanoma lines. *Ann. N.Y. Acad. Sci.*, **254**, 528.

Thomson, D. M. P., Ralich, J. E., Weatherhead, J. C., Friedlander, P., O'Connor, R., Grosser, N., Shuster, J. & Gold, P. (1978) Isolation of human tumour-specific antigens associated with $\beta_2$ microglobulin. *Br. J. Cancer*, **37**, 753.

Vanwijck, R., Bouillenne, C. & Malek-Mansour, S. (1975) Potentiation and arming of lymphocyte mediated immunity by sera from melanoma patients. *Eur. J. Cancer*, **11**, 267.

Viza, D. & Phillips, J. (1975) Identification of an antigen associated with malignant melanoma *Int. J. Cancer*, **16**, 312.

Warburg, O. & Christian, W. (1941) *Biochem. J.*, **310**, 384.